# The use of ultrasonography (US) in non traumatic musculo-skeletal disorders in infants and children

**DOI:** 10.1186/1824-7288-40-S1-A27

**Published:** 2014-08-11

**Authors:** Laura Tanturri de Horatio

**Affiliations:** 1 Department of Imaging, Bambino Gesù Children’s Hospital, IRCCS, Rome, Italy

## 

Ultrasongraphy (US) is a powerful diagnostic imaging tool for evaluating musculo-skeletal disorders in children. US is relatively cheap, non invasive and does not require sedation thus it is generally well-tolerated. Lack of ionizing radiation and dynamic imaging capabilities are significant advantages compared to CT and MRI too.

US is ideally suited to the evaluation of the bone, cartilage and soft-tissue structures, including tendons and ligaments, joints and muscles [1].

The combined use of new generation high frequency transducers and of Power Doppler increases the diagnostic accuracy of US.

US is widely used in the diagnosis of hip disorders in children [2]. In developmental displasia, it is essential for establishing an early diagnosis in order to allow a prompt treatment. In transient synovitis and hip infections US is able to demonstrate joint effusion, synovial thickening and cartilage damage. In Perthes disease US is a reliable technique to identify hip effusion, femoral head cartilage thickening as well as irregular, fragmented and flattened femoral epiphysis. In epiphysiolysis US allows the assessment of the severity of epiphyseal slipping by measuring the width of the physeal step [1].

Besides hip’s disorders, US can be employed in many other pathological conditions of the pediatric musculoskeletal system, including congenital, infectious, neoplastic and inflammatory disorders [3]. Particularly, in juvenile idiopathic arthritis (JIA) US is crucial for the diagnosis of the disease, the assessment of its severity and prognosis, the monitoring of disease progression and treatment response and the evaluation of complications related to the disease or its therapy. The US assessment of disease activity has been proven to be more informative than clinical examination. Moreover, multiple locations can be assessed during the same ultrasonographic session. US can guide intra-articular steroid injections for therapeutic purposes too.

In acute osteomyelitis US is able to identify early abnormalities in the soft tissues overlying the bone just few days after the onset of symptoms before the appearance of radiographic signs. As infection progresses, US can depict interruption of the cortical profile related to bone destruction and diffuse involvement of subcutaneous tissue with formation of abscesses.

Due to the peculiarities of the growing skeleton, the knowledge of musculoskeletal pediatric anatomy and especially of growth-related changes in healthy children is extremely important in establishing whether the US findings reflect pathology or are part of normal development [4].
